# Cone Beam Computed Tomographic Evaluation and Diagnosis of Mandibular First Molar with 6 Canals

**DOI:** 10.1155/2016/1016985

**Published:** 2016-01-21

**Authors:** Shiraz Pasha, Bathula Vimala Chaitanya, Kusum Valli Somisetty

**Affiliations:** Department of Conservative Dentistry and Endodontics, Sri Rajiv Gandhi College of Dental Sciences, Bangalore 560032, India

## Abstract

Root canal treatment of tooth with aberrant root canal morphology is very challenging. So thorough knowledge of both the external and internal anatomy of teeth is an important aspect of root canal treatment. With the advancement in technology it is imperative to use modern diagnostic tools such as magnification devices, CBCT, microscopes, and RVG to confirm the presence of these aberrant configurations. However, in everyday endodontic practice, clinicians have to treat teeth with atypical configurations for root canal treatment to be successful. This case report presents the management of a mandibular first molar with six root canals, four in mesial and two in distal root, and also emphasizes the use and importance of Cone Beam Computed Tomography (CBCT) as a diagnostic tool in endodontics.

## 1. Introduction

Precise study of the morphology of human teeth is required for the successful treatment with the objective of providing better oral health and restoring stomatognathic functions [1]. The mandibular first molar usually has 2 roots, occasionally 3. Further, there are generally 2 canals in the mesial root and one or 2 in the distal root. Vertucci and Williams [2] were the first persons to report the presence of middle mesial canal in the mandibular 1st molar and since then there were many case reports published showing the presence of mandibular molars with aberrant root canal morphology. In a radiographic study of extracted teeth Goel et al. reported that mandibular first molars had three mesial canals in 13.3% of specimens, four mesial canals in 3.3% specimens, and three distal canals in 1.7% of specimens [3]. It has been postulated that secondary dentin apposition during tooth maturation would form dentinal vertical partitions inside the root canal cavity creating root canals and the third root canal is also created by this process. Such third canals are situated mainly between the two main root canals, the buccal and lingual root canals [4].

This case report presents the management of the 1st mandibular molar with six root canals, four in mesial and two in distal root canal confirmed by CBCT.

## 2. Case Presentation

**Figure 1:**
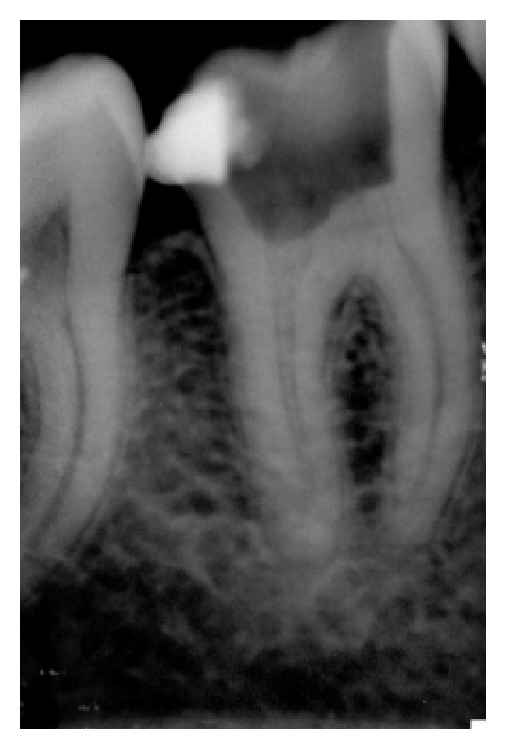
Pre-op X-ray.

**Figure 2:**
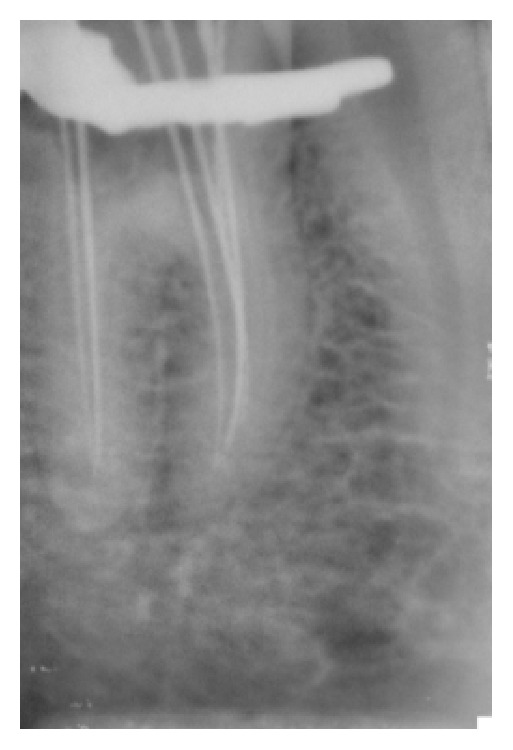
IOPA showing 4 mesial canals.

**Figure 3:**
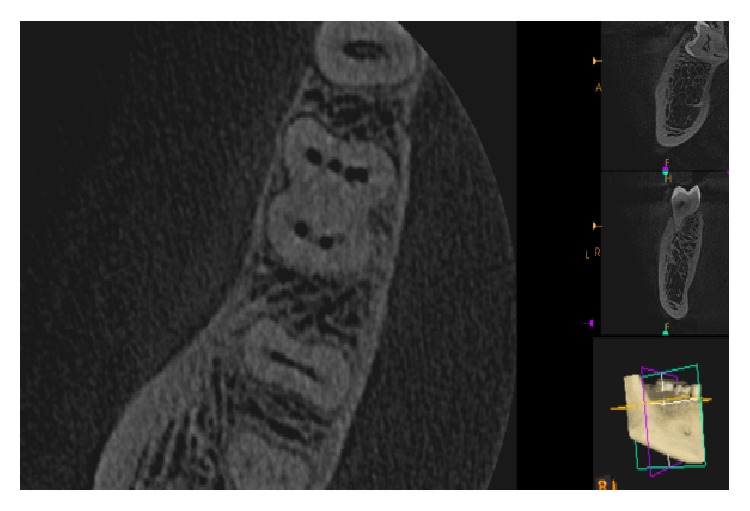
CBCT image showing 4 canals in mesial root.

**Figure 4:**
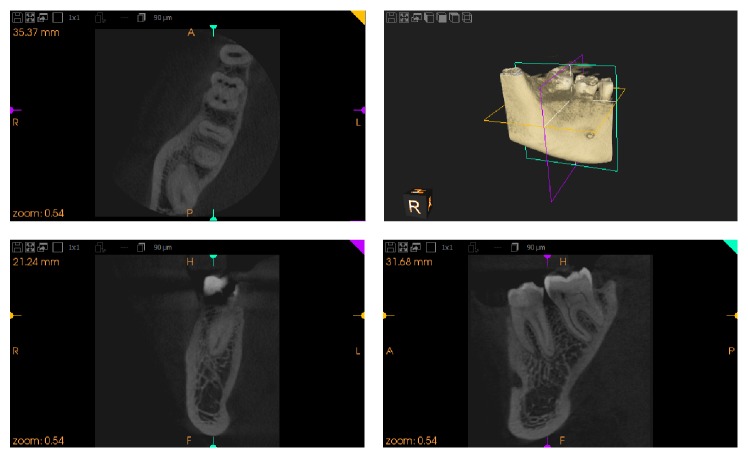
CBCT image showing 4 canals in mesial root.

**Figure 5:**
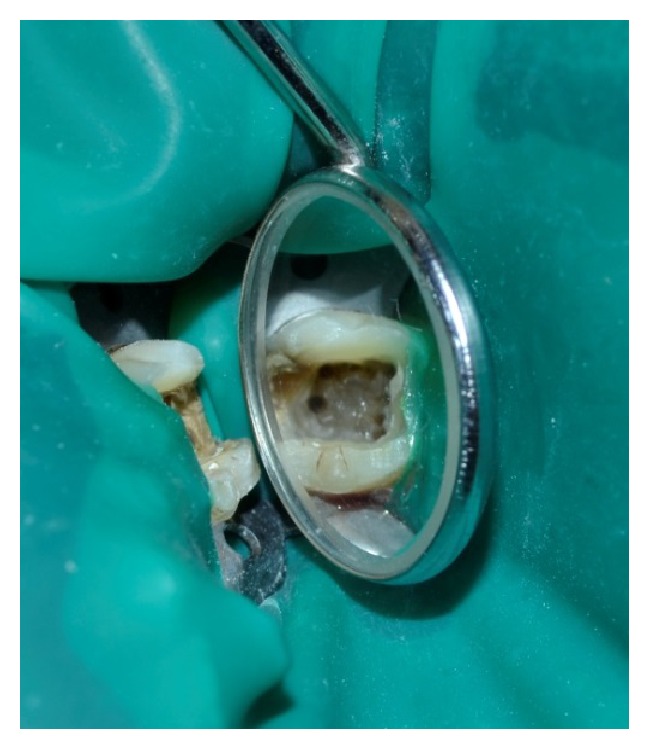
Intraoral image showing 6 canals.

**Figure 6:**
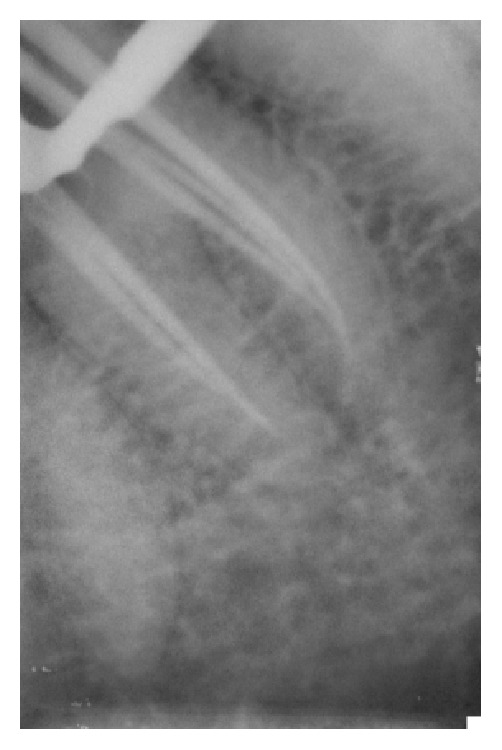
Master cone selection.

**Figure 7:**
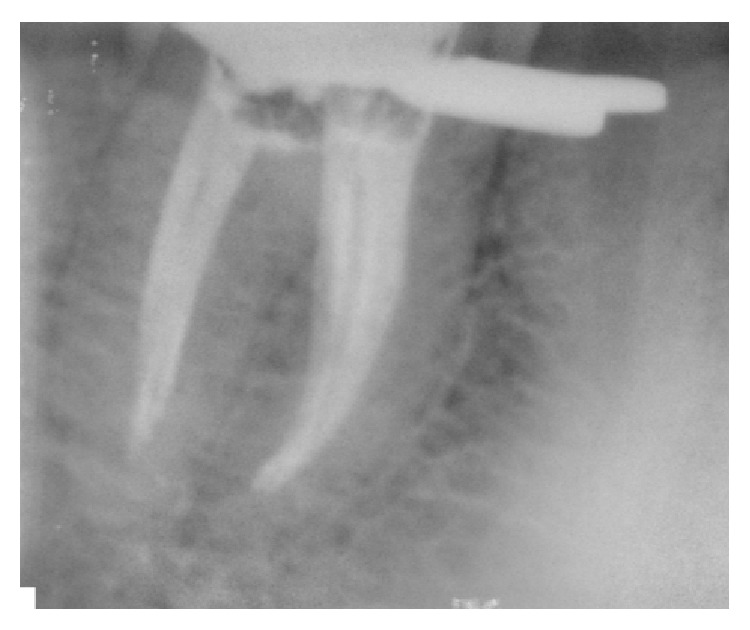
IOPAR after obturation.

A 30-year-old male patient with nonsignificant medical history reported to our department with a chief complaint of pain in right mandibular region. On history taking there were episodes of intermittent pain for the past 15 days. Pain was moderate in nature, nonradiating, aggravates on taking sweets and chewing foods, and relieves on taking medication. On clinical examination a deep carious lesion was seen with respect to 46. Exaggerated response was observed during pulp testing with electric pulp tester and lingering pain was observed with cold pulp test compared to contralateral teeth. IOPAR revealed radiolucency involving enamel, dentin, and pulp with no periapical changes in relation to 46 (Figure 1). It was diagnosed as acute irreversible pulpitis. Root canal treatment was decided and explained to the patient. After securing local anesthesia (2% lignocaine, inferior alveolar nerve block on the right side) rubber dam was applied and endodontic treatment was initiated. After gaining the proper access four canals were located, two in the mesial and two in the distal. It was evident under magnification that the MB and ML were placed well apart with an isthmus joining two canals. Hence, the possibility of MM canal should be anticipated in the isthmus. On exploration with DG-16 probe, we found 2 additional canals between MB and ML (Figure 5). IOPAR revealed one MM joining the MB canal and another joining the ML canal in the middle third. To confirm this we advised a CBCT of the right mandibular molar. CBCT revealed four canals in the mesial root and two canals in distal root (Figures 3 and 4). Access was refined and orifices were enlarged using orifice openers. The working length was determined with radiographic technique and apex locator (Figure 2). Both the mesial and distal canals were enlarged up to the size of 25/6% taper (M two, VDW) followed by an intracanal medication with calcium hydroxide and chlorhexidine was placed for 1 week. At the 3rd appointment, master cone was selected (Figure 6) and obturation was performed using cold lateral compaction technique and AH-plus root canal sealer.  Figure 7 shows the IOPAR immediately after obturation.

## 3. Discussion

The complicated and diverse root canal system poses a challenge to successful diagnosis and treatment. The incidence rates of MM canal are between 1 and 15% [5]. In most of the cases, middle/extra canals are hidden by a dentinal projection in the mesial and distal aspect of pulp chamber walls, and this dentinal growth is usually located between the two main canals. Pomeranz et al. [6] in their study found that about 12 out of 100 molars had MM canals. They classified them into Fin, confluent and independent. In a similar study done by de Pablo et al. [7] confirms the presence of MM canal in 2.6% mandibular first molars. Gulabivala et al. [8] described a four-canal pattern, but existing as two canals, in Burmese population. Newer diagnostic methods such as computerized tomography (CT) scanning greatly facilitate access to the internal root canal morphology. One of the most important advantages of CBCT is that operator can have a look at slices of tooth of interest [9]. Other diagnostic tools such as multiple preoperative radiographs, use of sharp explorer, ultrasonic tips, staining the chamber floor with 1% methylene blue dye, performing the sodium hypochlorite “champagne bubble test,” and visualizing canal bleeding points which are all important aids in locating root canal orifices are used to find out the additional canals present [5]. Also the use of operating microscope has revolutionized the practice of endodontics by allowing the clinicians to visualize the canal more efficiently [10]. Suspicion about a MM canal should always be anticipated when isthmus is clinically evident. The groove between MB and ML is a potential area to be addressed and the access should be modified for effective disinfection of root canal system. The clinicians should always suspect the possibility of additional canals in patients who are 40 years and above. Mandibular first molar with four canals in mesial root has been reported in literature thrice which makes our case report unique and worth mentioning to understand the complexity of root canal system of mandibular first molar.

## 4. Conclusion

Prognosis of the endodontic treatment on a long term is severely compromised due to the failure to locate and clean extra canals. This management is quite challenging. With good knowledge, the will to search, and the magnification and modern imaging techniques, the success rates can be improved. Our case report describes a successful management of a mandibular molar with 6 canals.

## References

[1] Bains R., Loomba K., Chandra A., Loomba A., Bains V. K., Garg A. (2009). The radix entomolaris: a case report. *Endodontic Practice Today*.

[2] Vertucci F. J., Williams R. G. (1974). Root canal anatomy of the mandibular first molar. *Journal of the New Jersey Dental Association*.

[3] Goel N. K., Gill K. S., Taneja J. R. (1991). Study of root canals configuration in mandibular first permanent molar. *Journal of the Indian Society of Pedodontics and Preventive Dentistry*.

[4] Martínez-Berná A., Badanelli P. (1985). Mandibular first molars with six root canals. *Journal of Endodontics*.

[5] Vertucci F. J. (2005). Root canal morphology and its relationship to endodontic procedures. *Endodontic Topics*.

[6] Pomeranz H. H., Eidelman D. L., Goldberg M. G. (1981). Treatment considerations of the middle mesial canal of mandibular first and second molars. *Journal of Endodontics*.

[7] de Pablo Ó. V., Estevez R., Sánchez M. P., Heilborn C., Cohenca N. (2010). Root anatomy and canal configuration of the permanent mandibular first molar: a systematic review. *Journal of Endodontics*.

[8] Gulabivala K., Aung T. H., Alavi A., Ng Y.-L. (2001). Root and canal morphology of Burmese mandibular molars. *International Endodontic Journal*.

[9] Barletta F. B., Dotto S. R., Reis M. D., Ferreira R., Travassos R. M. (2008). Mandibular molar with five root canals. *Australian Endodontic Journal*.

[10] Saunders W. P., Saunders E. M. (1997). Conventional endodontics and the operating microscope. *Dental Clinics of North America*.

